# The right time to quit: patients' experiences of smoking cessation support during COPD hospitalisation

**DOI:** 10.1080/21642850.2026.2704846

**Published:** 2026-08-11

**Authors:** Louise Muxoll Grønhaug, Jannie Christina Frølund, Anders Løkke, Ingeborg Farver-Vestergaard

**Affiliations:** a Department of Medicine, Lillebaelt Hospital, Vejle, Denmark; b Department of Regional Health Research, University of Southern Denmark, Odense, Denmark

**Keywords:** Nicotine dependence, addiction, chronic respiratory disease, inpatient care, smoking abstinence.

## Abstract

**Introduction:**

Smoking cessation is a central component of disease management in chronic obstructive pulmonary disease (COPD). Hospitalisation provides an opportunity to initiate smoking cessation support. The present study aimed to explore how patients with COPD perceived the usefulness of smoking cessation support in the context of hospitalisation.

**Methods:**

Patients receiving smoking cessation support during hospitalisation at the Department of Medicine, Lillebaelt Hospital, Denmark, were invited to semi-structured interviews via telephone one week after discharge. Interviews were transcribed verbatim and analysed inductively using qualitative content analysis.

**Results:**

Eight patients with COPD (mean age 73 years) were interviewed between January and March 2024. Patients described how their acute respiratory symptoms, the smoke-free environment and the easy access to pharmacological smoking cessation treatment during hospitalisation made smoking both impractical and unreasonable. A respectful, non-judgmental approach to smoking cessation support was emphasised as central to its perceived usefulness and acceptance. One week after discharge, seven patients remained abstinent and reported increased confidence in managing cravings with practical strategies and pharmacological smoking cessation treatment. However, many expressed uncertainty regarding long-term maintenance.

**Conclusions:**

Hospital-initiated smoking cessation support was perceived as timely and useful by patients with COPD. The combination of the hospital context and a respectful, person-centred approach facilitated early cessation. However, sustained abstinence after discharge was perceived to be challenging.

## 1. Background

Smoking cessation is essential to disease management in chronic obstructive pulmonary disease (COPD). Despite robust evidence that smoking cessation slows disease progression, improves symptoms and reduces mortality, approximately 35–40% of people with COPD continue to smoke (Global Initiative for Chronic Obstructive Lung Disease (GOLD), 2026; Nielsen et al., 2024; Tashkin, 2021; Wang et al., 2024). Smoking cessation is often more challenging for patients with COPD than for those without the disease, due to higher nicotine dependence, lower self-efficacy and high levels of psychological distress (Global Initiative for Chronic Obstructive Lung Disease (GOLD), 2026; Jiménez-Ruiz et al., 2015). Therefore, smoking cessation support should be tailored to this population's specific needs and circumstances with respect to both content and timing of delivery (Tashkin, 2021).

A recent Cochrane review demonstrated that smoking cessation interventions for hospitalised patients are most effective when behavioural counselling is combined with pharmacological smoking cessation treatment, such as nicotine replacement therapy (NRT), varenicline or cytisine (Streck et al., 2024). The review further showed that continued counselling after discharge improved rates of sustained abstinence. A clinical study found that respiratory inpatients had higher adherence to post-discharge cessation protocols and higher six-month quit rates than patients hospitalised for cardiac conditions, despite receiving identical in-hospital interventions (Garcia et al., 2018). This may suggest a stronger perceived link between illness and smoking specifically for patients with respiratory disease.

Despite strong evidence, smoking cessation support is inconsistently implemented in hospital care. In a large U.S. cohort, 58% of inpatients who smoked were never prescribed pharmacological smoking cessation treatment, and only 5% received cessation pharmacotherapy during admission or at discharge following their first hospitalisation (Nielsen et al., 2024). Similar challenges exist in Europe. In Denmark, brief smoking cessation advice during hospitalisation has been recommended for years, but most clinicians report that they do not routinely provide such support in practice (Jakobsen et al., 2025; Saei et al., 2025).

Several barriers limit the provision of smoking cessation support in hospital settings. These include limited time, insufficient knowledge among hospital-based healthcare professionals regarding cessation pharmacotherapy and behavioural interventions, and uncertainty about when and how to initiate cessation conversations with acutely ill or presumed unmotivated patients (Jørgensen et al., 2024; Malone et al., 2017; Russell et al., 2021; Sharpe et al., 2018).

Structured approaches may help overcome these barriers. We have previously demonstrated that it is feasible to implement a systematic approach to smoking cessation support for hospitalised patients with COPD – including training healthcare professionals to ensure they have sufficient time and competence to deliver the support (Farver-Vestergaard et al., 2025). In that study, the majority of patients reported abstinence after discharge. Compared with patients who did not sustain smoking cessation, abstinent patients also reported a lower symptom burden.

However, patients' experiences of hospital-initiated smoking cessation support remain underexplored. To accompany these findings and to improve interventions in the future, there is a need to better understand how patients with COPD perceive smoking cessation support provided during hospitalisation. Therefore, this study aimed to explore patients' perceived usefulness of tailored smoking cessation support during acute COPD-related hospitalisation (objective 1), and to explore how this support influenced their decision-making, motivation and perceived ability to initiate and sustain smoking cessation after discharge (objective 2).

## 2. Methods

### 2.1. Design

This qualitative study was part of a larger pilot study (Farver-Vestergaard et al., 2025). Semi-structured interviews were used to gain detailed insight into patients' experiences of smoking cessation support during hospitalisation. The present reporting of the study adheres to the Consolidated Criteria for Reporting Qualitative Research (COREQ) (Tong et al., 2007).

### 2.2. Setting and intervention

The intervention was integrated in routine inpatient care at the Department of Medicine, Lillebaelt Hospital, Vejle, Denmark. Smoking cessation support was delivered by a trained nurse as part of her usual work in the clinic. Sessions were brief and informal, with repeated and flexible contacts depending on patient's condition and length of stay. Before delivering the intervention, the nurse received training in evidence-based motivational and behavioural smoking cessation counselling as well as pharmacological smoking cessation treatment. All patients interested in quitting were offered pharmacological smoking cessation treatment free of charge during the hospitalisation based on their preference and the clinical appropriateness. This practice is not standard in Danish hospitals but had been implemented at the hospital where this study was conducted. Prolonged pharmacological treatment was provided by the hospital for the first couple of days after discharge, after which patients were required to purchase the medication themselves to continue treatment. Treatment decisions were made collaboratively, with input from a respiratory physician when needed. The nurse followed up on the patients' smoking status and needs via telephone at one and three months after discharge. Patients in need of further support were referred to community-based smoking cessation programmes. Supportive materials included a patient leaflet, an informational podcast, and a shared decision-making tool to facilitate personalised discussions about the advantages and disadvantages of smoking cessation.

### 2.3. Participants

Patients were recruited by the project nurse based on the following criteria:

Inclusion criteria:

1. Confirmed COPD diagnosis, based on spirometry according to GOLD guidelines (Global Initiative for Chronic Obstructive Lung Disease (GOLD), 2026)
2. Hospitalisation due to COPD exacerbation
3. Age ≥ 40 years
4. Active smoking at admission, based on patient self-report.

Exclusion criteria:

1. Inability to understand or communicate in Danish
2. Terminal illness or palliative care status

Purposive sampling ensured diversity in age, sex and length of hospitalisation (Moser & Korstjens, 2018). We aimed to recruit approximately ten patients to gather adequate and nuanced data to address our objectives. At recruitment, patients were informed that a follow-up interview would take place after hospital discharge.

### 2.4. Data collection

A semi-structured interview guide (see Supplementary material) was developed based on the study objectives and relevant literature (Kallio et al., 2016). The guide included open-ended questions on patients' a) previous cessation experiences, b) their perceptions of the smoking cessation support provided in hospital and c) their experiences managing cessation after discharge. The interview guide was pilot tested with two patients. No major adjustments were required and the two pilot interviews were included in the final dataset.

Patients were contacted by the interviewer via phone to schedule interviews in accordance with their preferences. Telephone interviews were used because they are cost-effective and suitable for elderly and frail individuals (Azad et al., 2021).

Interviews were scheduled one week after hospital discharge to reduce recall bias while still allowing patients to reflect on their initial experiences of managing smoking cessation at home. To ensure consistency, all interviews were conducted by the same researcher (LMG) (Dicicco-Bloom & Crabtree, 2006), a nurse and PhD student experienced in qualitative interviewing. As the interviewer had not been involved in delivering the smoking cessation support and was unfamiliar with its specific structure and content, she met with the project nurse before data collection to gain an overview of the intervention.

### 2.5. Data analysis

Interviews were audio-recorded and transcribed verbatim by the interviewer. The interviewer supplemented transcripts with written field notes, capturing immediate reflections on the interview situation (Phillippi & Lauderdale, 2018). Qualitative Content Analysis (QCA), was used to generate a structured understanding of patients' perspectives (Lyhne et al., 2025). No repeat interviews were carried out.

The analysis followed four steps: (Wang et al., 2024) establishing an overview of the material, (Nielsen et al., 2024) identification of meaning units, (Tashkin, 2021) condensation of meaning units into descriptive categories and (Global Initiative for Chronic Obstructive Lung Disease (GOLD), 2026) generation of explanatory themes (Lyhne et al., 2025). Preliminary analysis was conducted by LMG and IFV after the fifth interview to inform the initial coding. NVivo (Lumivero) software was used to code, extract and cluster meaning units. Coding and the analysis were conducted by LMG, with ongoing discussion and validation with IFV. As no new themes was identified in the final two interviews, sufficient information power was considered achieved (Malterud et al., 2016).

### 2.6. Ethical considerations

The study followed the principles of the Declaration of Helsinki (World Medical Association, 2024) and the Danish Code of Conduct for Research Integrity (The, 2025). The Regional Committees on Health Research Ethics for Southern Denmark waived the need for formal ethical approval for this project (S-20242000-127). The processing of personal data was notified to and approved by the Danish Data Protection Agency in the Region of Southern Denmark and the research aim and methods were preregistered in the internal record (No. 24/6720). Patients received both oral and written information from the smoking cessation nurse before providing their written consent. Confidentiality and patients' integrity were protected by using pseudonyms in all documentation.

## 3. Results

We invited ten patients to participate, and all initially agreed. One patient could not be reached, and another declined due to resuming smoking. Eight interviews were conducted between January and March 2024, 5–7 days after discharge. Interviews lasted on average 31 minutes (range: 12–43), excluding the introductory section; one interview ended prematurely when the patient chose to go outside for a cigarette.

The participating patients were older adults (mean age: 73 years) with COPD, hospitalised for acute respiratory infections, including six patients with pneumonia and two with influenza. There was substantial variation in the number of years since diagnosis; however, the mean duration since diagnosis was five years, with two patients having only recently received their diagnosis and two others requiring oxygen therapy. Length of hospital stay varied considerably, reflecting differences in clinical severity and recovery following infection (mean stay: 8 days). Patients also reported diverse smoking histories, with most having initiated regular smoking during adolescence or early adulthood, and with varying experiences of prior quit attempts. Patient characteristics are presented in Table 1.

**Table 1.** Characteristics of patients with COPD included in the interview study on perceived usefulness of smoking cessation support during hospitalisation.

ID	Sex	Age group	Education length	Living situation	Years since diagnosis	Admission length (days)	Previous quit attempts
1	Female	65–74	None	Alone	3–9	4–7	A couple
2	Female	75–84	None	Cohabiting	3–9	15–25	None
3	Male	75–84	Short	Cohabiting	>10	4–7	None
4	Male	75–84	None	Alone	<2	1–3	None
5	Female	75–84	Medium	Cohabiting	<2	4–7	None
6	Female	65–74	None	Alone	>10	8–14	Several
7	Male	55–64	None	Cohabiting	<2	8–14	A couple
8	Male	65–74	None	Alone	3–9	1–3	One

Notes: COPD (Chronic obstructive pulmonary disease). Data were self-reported during interviews, except for years since diagnosis and length of stay, which were provided by the nurse delivering the intervention. Data are reported in intervals to preserve anonymity of individual patients.

### 3.1. Main findings

All patients initiated smoking cessation during hospitalisation. Across interviews, patients described circumstances during hospitalisation as the immediate reason they initiated smoking cessation. They emphasised the importance of both the hospital context (where and when cessation was initiated) and the approach of the smoking cessation support (how it was offered) in shaping their decision and motivation to quit. For most, illness flare-up, enforced abstinence due to non-smoking in hospital and easy access to pharmacological smoking cessation treatment created a situation in which smoking became both impossible and unreasonable. Seven of eight patients expressed that, during hospitalisation, they gained a clear commitment or hope to remain smoke-free when they returned to their own home. However, one patient described resuming smoking shortly after discharge. The analysis yielded four themes aligned with the two objectives.

Objective 1 (experiences during hospitalisation) led to the themes:

1. A wake-up call to change: accepting support when smoking becomes impossible and unreasonable
2. It matters how they ask: engaging with support offered with kindness and respect
  Objective 2 (experiences after discharge) led to the themes:
3. Too foolish not to continue: committing to stay smoke-free at home
4. Feeling prepared but not fully free: the ongoing work of staying in control

**Figure 1.**
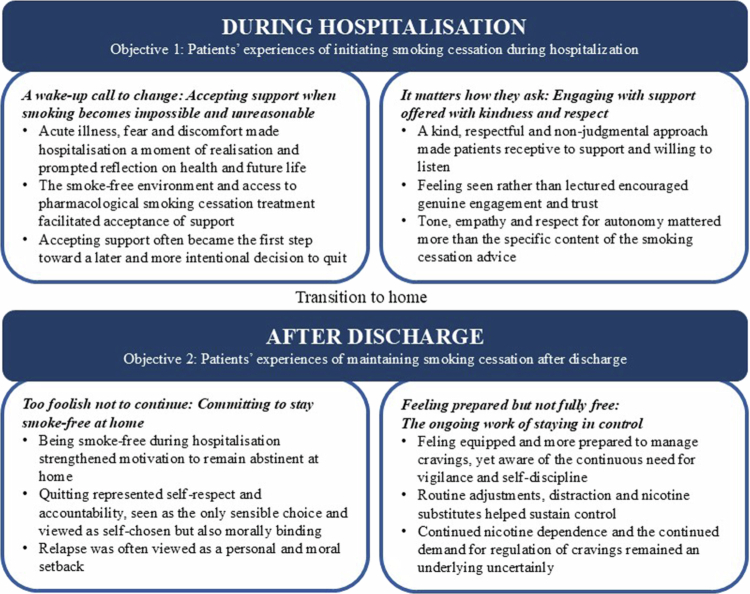
Patients' perceived usefulness of smoking cessation support in the context of hospitalisation due to chronic obstructive pulmonary disease (COPD). Findings from an interview study with 8 patients.

These themes illustrate a shift from externally enforced abstinence during hospitalisation to a more internalised, self-regulated commitment after discharge (Figure 1).

### 3.2. Theme 1. A wake-up call to change: accepting support when smoking becomes impossible and unreasonable

Hospitalisation was experienced both as a motivational ‘wake-up call’ and a practical barrier to smoking, which prompted patients to accept the offered smoking cessation support even when they were not yet fully committed to quitting. The experience of severe breathlessness and acute admission due to respiratory symptoms made smoking undesirable. Easy and immediate access to pharmacological smoking cessation treatment and behavioural support fostered reflection on health, vulnerability and the wish to prevent future readmissions. Patients described hospitalisation as the ‘right moment’ and a clear turning point to initiate change:

‘*It was just good timing, really. It just felt right, because I hadn’t smoked for a few days and didn’t have the energy or desire for it. You caught me at the right moment. (…) I somehow felt easier because I was already in the process, and also because, for all in the world, I don’t ever want to go through something like that again. I really didn’t feel like smoking while I was in there, not at all. (…) And I also have this sense that if you can stop thinking about it all the time, then you can stop smoking too.’* (ID 5)

Many patients experienced a major health scare in relation to the need for admission, e.g.

*‘I couldn’t breathe, so I thought, 'Now you’re quitting smoking, or else you'll die!' (…) Honestly, I truly thought I was dying. I was so frightened, absolutely terrified. (…) I’m not putting myself through that again. It would just be too stupid. So, I’m done.’* (ID 2).

This triggered reflection on family, aging and life priorities. Several patients emphasised wanting to stay healthy for their children or grandchildren, while others mentioned the economic relief of not spending money on cigarettes. Seeing other patients with advanced COPD served as a confronting image of what might lie ahead if they continued smoking:

‘​​​​​​*I saw all those COPD patients up there. It was a good advertisement for quitting smoking. That’s probably the biggest motivator. I don’t want to end up needing oxygen through my nose. (...) I think it was a mix of everything that got me started. But especially those COPD patients up there, they really scared me, for sure. I’ll never smoke regularly again. I won’t.’* (ID 8)

For most patients, this heightened awareness was inseparable from the hospital context itself. The smoke-free environment made smoking practically impossible, and patients thereby felt both compelled and supported to refrain from it. Accepting pharmacological smoking cessation treatment became the most natural and immediate solution to manage cravings in this setting:

*‘When you’re not doing well, I think you’re quicker to be motivated to say yes to something like that. That’s what I did, I said yes right away. (…) You can accept the patches* [pharmacological smoking cessation treatment], *and then just stop using them at home, if you still prefer to smoke. That's fine because then you can really decide for yourself once you get home.’* (ID 3)

The initial ‘yes’ to cessation support often stemmed from a pragmatic wish to reduce cravings rather than from firm conviction to quit smoking. However, most patients emphasised that the smoking-related conversations during hospitalisation helped them either to confirm a decision that had long been postponed or to consider cessation for the first time:

*‘…I felt ready to talk about it, as I realised it was necessary. It took being hospitalised first for me to be ready. (...) The talk was really the same as before, but it just felt different this time, maybe because I was paying more attention. (…) So I was happy to get the patches and talk about quitting. It was really good that they asked if I needed help.’* (ID 6)

### 3.3. Theme 2. It matters how they ask: engaging with support offered with kindness and respect

This theme highlights how a kind, respectful, non-judgmental approach encouraged patients to engage in smoking cessation support during hospitalisation. Patients consistently emphasised that *how* they were approached mattered more than *what* was said. They valued being treated with kindness and respect and having the opportunity to make an autonomous choice. When support was framed as a friendly and polite offer rather than a directive or moral lecture, patients felt motivated to accept help and participate actively in cessation efforts. Conversely, any form of pressure or moralising was described as counterproductive and likely to evoke resistance.

All patients recalled that the smoking cessation support was presented as an *offer*, which they could freely accept or decline:

*‘She (the nurse) approached me kindly, and it really matters how you approach and talk to people (...) If you are kind and willing to help, then I will do my best. I think it matters whether you decide to join in and say, yes, I want to stop smoking and get the help that can make it easier. It certainly shouldn't be with a wagging finger. (...) Then I just get stubborn and don't want to listen at all.’* (ID 1)

Several patients vividly contrasted this experience with more judgmental or moralising encounters from the past, which they found demotivating. For example, one said:

*‘It's important that she asks and makes the offer. (...) I think if someone had come and pressured me a bit, I would have just stopped and said ‘no!’. And then they wouldn't have been able to help me at all. (...) The least you can do is make it voluntary. So they don't just say 'yes' without meaning it, because then it won't make a difference if they just start right after they're out.’* (ID 7)

Most patients emphasised that they already knew smoking was harmful but appreciated when explanations were personalised to their individual health situation, such as their COPD and risk of readmission.

*‘But we need to be told how bad smoking is for COPD, like specifically why. When you understand it a bit more, you have more respect for it, and it helps with motivation. Because most of it you know already…’* (ID 8)

It is worth noting that many patients could not explicitly recall how these conversation started, how often they occurred or what the specific details of the information was. Only one patient mentioned discussing both advantages and disadvantages (ID 6), while others remembered visual aids (ID 1, ID 2). When asked about it by the interviewer, the patients attributed this memory lapse to either general poor memory or the stress and fatigue associated with being hospitalised, which often made it difficult to retain detailed information.

### 3.4. Theme 3. Too foolish not to continue: committing to stay smoke-free at home

Hospitalisation created a motivational momentum that supported patients' commitment to remain smoke-free after discharge. Having already endured the most difficult withdrawal period, most described it as unreasonable, or even ‘too foolish’ to resume smoking. They described continued abstinence as a matter of integrity and responsibility for preventing further self-inflicted harm. This was often intertwined with regret over past smoking and earlier missed opportunities to quit:

*‘It would be stupid now that I've already gotten through the worst, the cravings and all that. And I've kind of figured out a way, with the lozenges* [pharmacological smoking cessation treatment] *and such, to get through them. So it would be really stupid to give in again. It's stupid enough that I didn't pull myself together earlier.’* (ID 6)

Many described that the ‘real’ decision or commitment to stay smoke-free was made almost immediately after discharge. It was described as a natural continuation of what they had already achieved during hospitalisation. Several described that by the time they returned home, they were already ‘in the process’ of quitting and only needed to decide to *continue* quitting, which appeared as an easier decision than the decision to *initiate* quitting. Several patients used phrases like ‘holding on’ or ‘keeping at it’. They did not want to ‘throw away’ the progress made:

*‘It's probably a bit easier because you're already in the process. So now, it’s just about continuing.’* (ID 3)

In contrast, one participant resumed smoking shortly after returning home:

*‘It’s the only thing I have left. (…) At my age, it doesn’t really matter anymore. The only thing I enjoy when I get up in the morning, before I go to bed, and with my cup of coffee is having a smoke. So that’s what I’m sticking with. Otherwise, nothing really matters.’* (ID 4)

His account illustrates that the motivational shift experienced by most patients, where remaining smoke-free felt like the only sensible choice, was not universal. For some, the addiction, emotional meaning and daily comfort associated with smoking outweighed both health concerns and the practical reasoning that supported others.

### 3.5. Theme 4. Feeling prepared but not fully free: the ongoing work of staying in control

The first week at home required ongoing effort and practical adaptation to sustain cessation. Although hospitalisation had helped patients through the most intense cravings and equipped them with pharmacological smoking cessation treatment and coping strategies, staying smoke-free demanded daily self-regulation. Cravings were generally described as sudden but brief and more manageable than expected, yet they still required attention and planning. Patients increasingly drew on distraction, routine adjustments and avoidance of triggers to maintain a sense of control. Several were unsure whether these strategies had been explicitly discussed during hospitalisation or whether they emerged intuitively once home. Anyhow, these strategies strengthened patients’ sense of control and their confidence in being able to manage cravings.

Patients adjusted their routines to reduce exposure to triggers. Several avoided specific rooms (e.g. the kitchen or balcony), certain times of day or established habits associated with smoking (e.g. drinking coffee):

‘​​​​​*I told my wife, ‘You know what, I shouldn’t go into the kitchen,’ because that’s when I get the craving. But now, it doesn’t matter. I can go into the kitchen and walk around. I don’t get the urge to smoke anymore. I’m surprised at how easy it’s become (…) This time, I can feel it in my body and in my thoughts. Something has changed, and I know it will last.*’ (ID 7)

For most patients, pharmacological smoking cessation treatment played a central role. Many described how patches and lozenges [nicotine substitutes] ‘pushed cravings into the background’ or ‘kept them steady’, enabling them to stay in control. For some, they were experienced as reassuring tools; for others, they were reminders of ongoing nicotine dependence. A few were frustrated about not feeling entirely ‘finished’ with nicotine:

*‘I’m not really done, because I’m dependent on these other things* [nicotine substitutes] *now. They’re better for you, but it’s frustrating not to be completely done. Addiction is a tricky thing, something I don’t fully trust. I don’t feel finished until those aids are off the table. (…) I’m doing what I can not to slip up again, and told myself that I’m the one in charge, that I’m done with it. Now I just need to quit completely.’* (ID 8)

Several described this ambivalence of feeling prepared but not fully free. Patients expressed confidence because cravings were manageable, but they also described an underlying uncertainty about whether difficulties with sustained abstinence could return:

*‘It’s easier now because I’ve realised how quickly the cravings pass. But, you never know if it will suddenly get harder to stay quit, you know. I’ve told myself that this is it, but there’s still a little doubt about whether I can manage. You always hear it’s so hard, and when you don’t find it hard, you start to wonder, ‘will it suddenly get difficult?’’* (ID 6).

## 4. Discussion

Patients in the present study perceived the smoking cessation support offered during admission as both useful and well timed. Acceptance and usefulness of support was strongly influenced by *how* it was offered: a kind, respectful and non-judgmental approach made patients receptive to pharmacological smoking cessation treatment and behavioural counselling. After discharge, most patients felt better prepared to manage cravings. They attributed this to the combination of hospitalisation, access to pharmacological smoking cessation treatment and practical strategies such as distraction and avoiding triggers. Findings indicated a gradual shift from externally imposed abstinence at the time of admission to internalised self-regulation and intrinsic motivation at home. However, many patients experienced lingering uncertainty, continued nicotine dependence and a sense of loss related to non-smoking. This illustrates the challenges of sustaining cessation beyond the acute setting.

### 4.1. Support during hospitalisation

The COM-B behaviour change model – referring to Capability, Opportunity, Motivation and Behaviour - can be used for a broader contextualisation of the present study findings (Michie et al., 2011). The component of ‘Motivation’ refers to the internal drive to perform a behaviour, including beliefs and emotional impulses. Patients in the present study consistently described the setting of the hospital admission as a decisive moment where acute symptoms, such as breathlessness and the resulting chock and anxiety, made smoking feel unreasonable and even dangerous. While this belief had existed for many patients also prior to the hospitalisation, a vivid emotional tone was suddenly added to it, which underscored the relevance of quitting. This context-related change in motivation has also been described as a ‘teachable moment’ (Dohnke et al., 2012; Jiménez-Ruiz et al., 2015; Taylor et al., 2007). For example, a recent study of patients with asthma and COPD using Belgian health registry data (Vauterin et al., 2025) found that not only stroke and acute myocardial infarction, but also exacerbations and spirometry testing were associated with a significantly increased likelihood of attempting smoking cessation. However, as highlighted by Lang in an editorial in CHEST (Lang, 2023), it is important that smoking cessation support is not based solely on patients' immediate willingness to quit. Nicotine dependence is more than a behaviour or state of mind. Although many healthcare providers offer cessation pharmacotherapy only to patients who express readiness to quit, such treatment should be considered standard care for all patients with tobacco use disorder, regardless of comorbid conditions (e.g. COPD, cardiac disease, lung cancer). Hence, any such encounter represent a *treatable* moment, not only a *teachable* one (Lang, 2023), across diseases and in outpatient as well as inpatient settings. ‘Opportunity’ in the COM-B model refers to the physical (e.g. resources and location) and social (e.g. cultural norms and social support) environment that enables or hinders the behaviour. In the present study, patients described how the restricted availability of cigarettes and smoking areas in the hospital, along with the immediate access to pharmacological smoking cessation treatment, made it easier than ever before to get started on the cessation process. This finding aligns with the concept of ‘opportunistic cessation interventions’ (Steinberg, 2026) which refers to opportunities to address tobacco use when individuals who smoke present in settings where smoking cessation services can be offered – rather than requiring them to actively seek out such services themselves. The last component of ‘Capability’ refers to the physical and psychological capacity to perform the behaviour, including knowledge, skills and feelings of strength and self-efficacy. In this regard, patients in the present study expressed the importances of receiving information specifically on how smoking impacts their COPD condition and their future health risks. It was also highlighted that when support was offered respectfully and framed as voluntary, patients perceived it as useful and were more likely to engage. Another recent study (Kacmarek et al., 2026) indicated that patients who are interested in quitting smoking often perceive healthcare providers as unable or unwilling to offer effective support, either because smoking cessation is not being seen as part of their health providers' role or due to a perception of providers' having low expectations of patient success. In our study, when patients' became aware that they had already started on the cessation process with support during the hospitalisation, they realised their own strength and skills to continue the process.

Our findings also align with an existing integrative review of qualitative studies across disease populations, which demonstrates that hospitalisation is perceived by both patients and healthcare professionals as an appropriate setting and time to address smoking (Epton et al., 2023). The review recommends the use of a programme champion, which was indeed a feature of the clinical setup in the present study. The review also highlights the need for ongoing education of healthcare professionals delivering the intervention. As the present study only included one trained nurse delivering the intervention, and as sustainability and fidelity of the intervention were not assessed over time, we are unable to draw conclusions regarding the intervention's wider integration in the clinic. Moreover, existing studies indicate that the availability, cost and beliefs about pharmacological smoking cessation treatments are associated with the adherence to – and thereby the effectiveness of – these treatments (Pacek et al., 2018). This is consistent with our findings, in which the free availability and guided administration of such treatment served as an important enabler of the smoking cessation process, according to the patients.

The findings of the present study highlight that a respectful and engaging communication style was a key enabler of initiating the smoking cessation process during hospitalisation. This is consistent with both the shared decision-making framework (Chambers, 2023) and the motivational interviewing framework (Rollnick & Miller, 1995), which also formed the basis of the nurse's training for delivering the intervention. According to the Stages of Change, healthcare professionals should apply different communicative techniques and approaches depending on whether the patient is in the contemplation, preparation, action or maintenance stage of the change process (DiClemente et al., 1991; Prochaska & Diclemente, 1986). The flexible and patient-centred delivery of the intervention in the present study appears to have facilitated patients' experiences of feeling acknowledged, respected and supported. Rather than delivering a fixed intervention with predetermined session duration and intervals, the nurse – who was a familiar member of the inpatient staff – was able to integrate smoking cessation support into patients' routine care during hospitalisation. The effectiveness of this flexible, integrated approach compared with more standardised intervention formats warrants investigation in future trials.

### 4.2. Cessation maintenance after hospital discharge

While patients in the present study generally described becoming well-prepared for cessation maintenance during the hospitalisation, they also described that sustaining abstinence in the week after discharge was experienced as ongoing work with management of cravings and practical self-regulation. Some also described a sense of not being ‘fully free’ while using nicotine substitutes, as they knew they were still nicotine dependent.

In the present study setup, the nurse delivering the intervention during hospitalisation conducted follow-up telephone calls at one and three months after discharge to support patients' smoking cessation efforts. The study findings suggest that these follow-up intervals may be too long, as patients described the transition to their home environment – where habitual cues and triggers are re-established – as particularly challenging. Supporting this observation, a recent review of randomised controlled trials of smoking cessation interventions in COPD reported substantial heterogeneity in intervention intensity, duration and components, with only a minority of studies achieving long-term abstinence (Jakobsen et al., 2025). Nonetheless, two high-performing studies were distinguished by approaches characterised by frequent, brief contacts over an extended period following smoking cessation initiation (Lou et al., 2013; Sundblad et al., 2008). Numerous other studies further support the value of structured follow-up processes and continued contact (Streck et al., 2024; van Eerd et al., 2016).

The results of the present study also support the well-known issue that nicotine replacement therapy might only postpone, not solve, the problem, as patients using these substances are still addicted to nicotine, and thereby may be at high risk of relapse to smoking (Tashkin, 2021). Other medical options may therefore be more favourable, e.g. varenicline or cytisine, but independent on what medical compound is offered, follow-up is needed to ensure continued abstinence and patient self-efficacy.

When interviewed approximately one week after hospital discharge, the majority of patients in the present study were unable to recall the specific content of the smoking cessation support, aside from being approached in a kind and non-judgmental manner. This finding is consistent with previous studies indicating that hospitalisation is often characterised by instability, psychological distress and disruption of normal routines, which can compromise patients' decision-making and capacity to process and retain information (John et al., 2020; Sommer et al., 2018). This further highlights the need for follow-up support once patients have regained stability and routine in their home environment. Moreover, the findings underscore the importance of supplementing verbally delivered information with written or audio–written materials that the patients and their caregivers can re-access at other times, which is also seen more generally when it comes to recall of relevant information in healthcare (Frølund et al., 2024). A systematic review tentatively suggests that written decision aids may improve smoking cessation knowledge and decisional quality as well as increase quit attempts (Moyo et al., 2018). However, evidence regarding their effectiveness in achieving long-term, sustained smoking abstinence remains inconclusive, indicating a need for further research in this area. Taken together, the existing literature suggests that smoking cessation is influenced not only by the intervention components themselves, but also by how and when support is delivered.

### 4.3. Clinical implications

The clinical benefits of smoking cessation in patients with COPD include improvements in lung function, breathlessness and other respiratory symptoms, reduced exacerbation risk, enhanced exercise capacity and lower mortality (Nielsen et al., 2024; Wang et al., 2024). Therefore, smoking cessation should be regarded not merely as a lifestyle modification in COPD, but as an effective, evidence-based treatment that ought to be offered alongside other core components of COPD management. Based on the findings of the present study, a number of resource requirements and potential barriers should be considered when scaling up such an intervention. First, pharmacological cessation treatment was described as a central component by patients in this study. At the study site, this treatment was provided free of charge, highlighting the need to consider cost implications in other settings. Second, the manner in which healthcare providers communicate with patients about smoking cessation appeared highly influential for patients in the present study. Although the nurse delivering the intervention was trained in motivational interviewing, individual communication style may also have contributed to patient engagement. Variability in communication skills and training among healthcare providers should therefore be considered as a potential barrier to implementation. Third, patients reported feeling supported by the follow-up calls from the hospital nurse. When scaling up the intervention, the additional demands on staff time and resources associated with such follow-up should be taken into account.

Nonetheless, findings from the present study, together with existing literature, underscore the clinical relevance and patient-perceived meaningfulness of delivering smoking cessation support as an integrated component of inpatient care. For example, a recent scoping review (Jiang et al., 2024) synthesising 53 trials demonstrated that nurses often play a central role in smoking cessation interventions. Most commonly, they serve as assessors, educators and/or practice facilitators, often by applying motivational interviewing and the 5 A method – Ask, Advice, Assess, Assist, Arrange – which is also the framework recommended in the international COPD guidelines (GOLD) (Global Initiative for Chronic Obstructive Lung Disease (GOLD), 2026). However, multidisciplinary care is needed, and the review also highlighted a need for enhanced and structured training programmes to support the effective delivery of smoking cessation support.

As previously noted, several barriers must be overcome before smoking cessation support can be effectively integrated in routine clinical practice. Similar to patients, who require sufficient capability, opportunity and motivation to change their smoking behaviour, healthcare professionals also need these components to modify and sustain their smoking cessation support practices (Dyson & Cowdell, 2021). Therefore, when scaling up interventions such as the one evaluated in the present study, it is essential to consider the COM-B model from the perspective of healthcare professionals and to conduct comprehensive evaluations of implementation processes and outcomes. This can be achieved, for example, by applying the more detailed Theoretical Domains Framework (TDF) (Cane et al., 2012) in combination with the broader Consolidated Framework for Implementation Research (CFIR) (Damschroder et al., 2009). While the COM-B and TDF focuses mainly on individual determinants of behaviour change, CFIR additionally addresses organisational structures and the external context in which healthcare organisations operate. Furthermore, given patient preferences for accessible support and input from peers, hybrid or telehealth models may extent the in-hospital interventions to provide timely and convenient support at home during cravings (Tregobov et al., 2024).

### 4.4. Strengths and limitations

This study provides in-depth insights into patients' experiences of hospital-based smoking cessation support. The semi-structured interviews were conducted shortly after discharge, which allowed for as good recollection as possible during a period of distress and instability.

However, several limitations should be considered when interpreting the findings. First, participants were recruited from a single medical ward at one hospital in Denmark, and the intervention was delivered by only one nurse. This limits the applicability of the findings to other clinical settings and healthcare systems, as well as to intervention models involving multiple providers with varying levels of experience and training. Second, the study included only patients who accepted smoking cessation support during hospitalisation. Patients who declined the intervention were not approached, which may have limited insight into the perspectives and needs of those who are less motivated or more resistant to smoking cessation support. Consequently, the findings primarily reflect the experiences of patients who were at least initially receptive to support. Third, inclusion of actively smoking patients was based on self-report, which may have introduced misclassification bias due to potential underreporting of smoking status. However, patients with nicotine dependence are thought to be less likely to underreport in an inpatient setting, as enforced smoking restrictions would trigger withdrawal symptoms and cravings that are difficult to conceal. Alternatively, the use of biochemical verification, such as cotinine measurement in urine samples, could also introduce bias, as it may adversely affect the patient-interviewer relationship and potentially reduce patients' willingness to disclose their genuine experience with smoking cessation support. Fourth, patients frequently reported difficulty recalling specific components of the intervention and events that occurred during hospitalisation. This challenge may reflect the broader cognitive and emotional burden associated with acute illness and hospital admission and may also have affected the depth of the interview data. While alternative methods such as experiential sampling or ecological momentary assessment could potentially enhance data richness, these approaches may be less feasible in acutely ill, vulnerable populations with reduced cognitive capacity. Finally, as post-discharge interviews may have influenced patients' continued motivation and behaviour, alternative data collection methods – such as participatory or observational data collection during hospitalisation – could have provided valuable contextual insights into how smoking cessation support was delivered and received in real time. However, such methods were not feasible within the present study due to the substantial resource requirements associated with continuous observational data collection in an inpatient setting.

## 5. Conclusions

This interview study indicates that hospitalisation represents a clinically meaningful context for initiating smoking cessation in patients with COPD. Acute exacerbation of illness, enforced abstinence due to prohibited smoking in hospital and immediate access to pharmacological smoking cessation treatment facilitated early engagement. The respectful, person-centred approach enhanced patients' acceptance and motivation. Although patients gained confidence in managing cravings during hospitalisation, maintaining abstinence after discharge was perceived to be challenging due to ongoing nicotine dependence.

## Supplementary Material

Supplementary Material Supplementary material Interviewguide.docx

## Data Availability

The data that support the findings of this study are available on request from the corresponding author, IFV. The data are not publicly available due to their containing information that could compromise the privacy of research participants.

## Generative artificial intelligence (AI)

The authors used ChatGPT (OpenAI, GPT-5.2) was used to assist with language editing and grammar checking. The authors reviewed and approved all content and take full responsibility for the final manuscript.
