# Supplementary material for: The right time to quit: patients' experiences of smoking cessation support during COPD hospitalisation
Source: Health Psychol Behav Med. 2026 Aug 11;14(1):2704846. doi: 10.1080/21642850.2026.2704846 (PMC13463439; doi:10.1080/21642850.2026.2704846)
Supplement: Supplementary file 1 [file RHPB_A_2704846_SM8006.docx]

# Supplementary material: Interview guide

This semi-structured interview guide was developed to explore experiences and perceptions of smoking cessation support received during hospitalization among patients with chronic obstructive pulmonary disease (COPD). The guide was designed to capture patients’ reflections on both the content and delivery of smoking cessation support, as well as their experiences following discharge. Supplementary probing questions were used as needed to encourage elaboration and to deepen participants’ responses.

**Note:** The interview guide was originally developed in Danish. This English version is provided to enhance transparency and to allow readers to understand and assess the empirical foundation of the analysis.

**Introduction**

- Brief introduction of the interviewer
- Purpose of the interview study
- Your participation: e.g. time commitment, format, voluntariness, confidentiality, and audio recording
- Confirmation of continued willingness to participate

**Living with COPD**

You received support for smoking cessation during your hospitalization. We will return to that shortly. First, I would like to learn a bit more about you and your everyday life.

- Could you briefly tell me about yourself? (Age, education, employment, family situation)
- How many years ago were you told that you had COPD? (Diagnosis by general practitioner/ hospital?)
- What was the reason for your hospitalization?
- What thoughts did you have about quitting smoking before you were hospitalized?
  - How many times have you previously tried to quit smoking?

**Smoking cessation support during hospitalization**

Now I would like you to think back to your hospital stay, particularly the occasions when smoking was discussed with the nurse and how you received support to quit smoking.

**The conversations**

1. How did the conversation about your smoking first come up with the nurse?
2. Can you describe the conversation you had with the nurse when you decided that you wanted help to quit smoking?
   - Do you remember what the nurse said or did?
3. What information did you receive about smoking (and about the impact of smoking on your health)?
   - What were you told about the advantages and disadvantages of smoking and smoking cessation?
   - How did this information affect you?

**Other Interventions**

4. Do you remember what else was done to help you remain smoke-free while you were hospitalized?
   - Did you receive any medical treatment? (e.g., nicotine replacement therapy)
   - Did you receive any other type of support or follow-up?

5. What was your first thought when the nurse started talking to you about smoking?
   - What did you think and feel during the conversation (e.g., about the benefits of quitting smoking)?
6. When you recall the conversations about smoking and the support you received for smoking cessation, how did these differ from previous occasions when you discussed smoking cessation with a nurse or a physician?
   - Was there anything about the conversations that surprised you or seemed unexpected?
   - Was there anything from the support you received that you remember particularly clearly or made a strong impression on you?
7. What do you think about being offered help to quit smoking while you were hospitalized?
   - What significance did the timing and setting have for your motivation to quit smoking?
8. What led you to decide that you wanted to quit smoking?
   - What was particularly helpful or supportive?
   - Was there anything that could have been done differently or better?
9. Was there any type of support or follow-up that you missed or felt you needed while you were hospitalized?

**Smoking cessation support after discharge – At home**

Now I would like to ask about your experiences with smoking cessation after you returned home.

10. Have you smoked since you returned home from the hospital? (if not already mentioned)
    - What has been decisive for you in relation to:
      - (No) maintaining smoking cessation?
      - (Yes) starting to smoke again? (same level as before?)

Would you like to receive further support to quit smoking?

11. Was there any type of support or follow-up that you missed after returning home?
    - Was there anything that would have made it easier for you to maintain smoking cessation?
12. In the period after you returned home:
    - Has the municipality contacted you regarding further smoking cessation support?
      - What are your expectations of this support?
    - Have you had follow-up appointments with your general practitioner (e.g., regarding medication)?

**Evaluation of the overall smoking cessation support**

13. Overall, how would you evaluate the smoking cessation support you received?
    - What was the most important thing you gained from accepting the offer?
    - Was there anything that you found particularly helpful or that you felt was lacking?

**CLOSING**

14. Is there anything we have not discussed that you think is important to mention?
15. How was your experience of participating in this interview?
